# The Application of Data Envelopment Analysis to Emergency Departments and Management of Emergency Conditions: A Narrative Review

**DOI:** 10.3390/healthcare11182541

**Published:** 2023-09-14

**Authors:** Mirpouya Mirmozaffari, Noreen Kamal

**Affiliations:** Department of Industrial Engineering, Dalhousie University, 5269 Morris Street, Halifax, NS B3H 4R2, Canada; noreen.kamal@dal.ca

**Keywords:** data envelopment analysis, emergency department operations, acute management of stroke, stroke patients, emergency transfer to a tertiary hospital, acute stroke treatment, acute myocardial infarction treatment, endovascular thrombectomy, percutaneous coronary intervention

## Abstract

The healthcare industry is one application for data envelopment analysis (DEA) that can have significant benefits for standardizing health service delivery. This narrative review focuses on the application of DEA in emergency departments (EDs) and the management of emergency conditions such as acute ischemic stroke and acute myocardial infarction (AMI). This includes benchmarking the proportion of patients that receive treatment for these emergency conditions. The most frequent primary areas of study motivating work in DEA, EDs and management of emergency conditions including acute management of stroke are sorted into five distinct clusters in this study: (1) using basic DEA models for efficiency analysis in EDs, i.e., applying variable return to scale (VRS), or constant return to scale (CRS) to ED operations; (2) combining advanced and basic DEA approaches in EDs, i.e., applying super-efficiency with basic DEA or advanced DEA approaches such as additive model (ADD) and slack-based measurement (SBM) to clarify the dynamic aspects of ED efficiency throughout the duration of a first-aid program for AMI or heart attack; (3) applying DEA time series models in EDs like the early use of thrombolysis and percutaneous coronary intervention (PCI) in AMI treatment, and endovascular thrombectomy (EVT) in acute ischemic stroke treatment, i.e., using window analysis and Malmquist productivity index (MPI) to benchmark the performance of EDs over time; (4) integrating other approaches with DEA in EDs, i.e., combining simulations, machine learning (ML), multi-criteria decision analysis (MCDM) by DEA to reduce patient waiting times, and futile transfers; and (5) applying various DEA models for the management of acute ischemic stroke, i.e., using DEA to increase the number of eligible acute ischemic stroke patients receiving EVT and other medical ischemic stroke treatment in the form of thrombolysis (alteplase and now Tenecteplase). We thoroughly assess the methodological basis of the papers, offering detailed explanations regarding the applied models, selected inputs and outputs, and all relevant methodologies. In conclusion, we explore several ways to enhance DEA’s status, transforming it from a mere technical application into a strong methodology that can be utilized by healthcare managers and decision-makers.

## 1. Introduction

Healthcare systems worldwide are under pressure to provide timely access to urgent conditions in emergency departments (EDs) as wait times continue to increase [1,2], creating greater demands to enhance their efficiency. However, assessing the effectiveness of EDs is a complex task that should not be underestimated. An ED serves as the primary entry point to a hospital and plays a crucial role in hospital management. Due to the constant and unscheduled arrival of patients, the ED often experiences high levels of overload, leading to long waiting times for patients. This has a significant impact on patients suffering from conditions such as stroke and myocardial infarction, which require immediate treatment in the ED [3,4]. Therefore, EDs must be evaluated for their efficiency.

The potential tools to evaluate the efficiency of EDs include data envelopment analysis (DEA). The fundamental concept of DEA is to identify an optimal performance frontier comprising efficient decision-making units (DMUs) that cover all the ineffective DMUs. The efficiency value for each DMU can be determined by measuring its deviation or gap between a point and the frontier. Over the past decades, DEA has progressively been applied to EDs, proving its suitability in this area. DEA possesses various characteristics that make it an appealing instrument for evaluating the performance of EDs. Its ability to proficiently oversee numerous resources in an ED and monitor health results throughout the process of change is a notable benefit. EDs are typically the primary entry point for hospital admission. Because of the accidental nature of patients attending, the department should provide initial care for various diseases and injuries, some of which could be potentially life-threatening and need urgent care. EDs of most health centers operate 24 h a day 7 days a week, although supervision levels may differ to reflect patient volume.

Bridging the gap between evidence and practice concerning ED efficiency analysis is essential. Further research is needed to tackle methodological challenges in implementing efficiency analysis for EDs by managing emergency conditions effectively and enhancing the availability of valuable evidence. This article aims to be an initial step in increasing our understanding of how DEA is applied in EDs to assist decision-makers in increasing the number of eligible patients who receive advanced treatments for conditions such as acute ischemic stroke and acute myocardial infarction (AMI), including the urgent transfer of these patients to receive the treatment. Subsequently, we provide further details regarding acute ischemic stroke treatment and acute myocardial infarction treatment.

Ischemic stroke is the most common type of stroke, accounting for roughly 85% of all instances of strokes. Fortunately, medical treatment in the form of thrombolysis (alteplase and now Tenecteplase) offers a viable treatment for ischemic stroke. Alteplase, also known as tissue plasminogen activator (tPA), is a medication used for its fibrinolytic properties. Thrombolysis, typically using Alteplase, is an established and commonly employed treatment for ischemic stroke. Tenecteplase, a genetically modified derivative of Alteplase, offers potential advantages compared to Alteplase, including improved restoration of blood flow and a reduced risk of bleeding. A pivotal randomized controlled trial (known as the national institute of neurological disorders and stroke (NINDS) trial) revealed that 39% of individuals treated with alteplase exhibited no disability, as opposed to 26% of untreated patients [5]. Around 25% of individuals afflicted by ischemic stroke can be suitably managed with thrombolysis. Consequently, healthcare facilities equipped with a computed tomography (CT) scanner and specialized stroke treatment capabilities widely provide thrombolysis therapy. In 2015, a series of randomized controlled trials [6], including a trial comparing EVT with conventional treatment for patients with acute ischemic stroke, the ESCAPE trial led by Canadian researchers [7], demonstrated the remarkable effectiveness of a novel treatment for ischemic stroke patients: endovascular thrombectomy (EVT). This procedure involves the mechanical removal of blood clots using devices like stent retrievers and/or aspiration techniques [8]. EVT is reserved for patients afflicted by a severe form of ischemic stroke known as large vessel occlusion (LVO), which accounts for around 30–40% of all ischemic stroke cases [9]. The outcomes of EVT are impressive: approximately 26.9% of stroke patients treated with EVT experience a full recovery without any disability, in contrast to 12.9% of patients who do not receive EVT. Furthermore, 46.0% of EVT-treated patients have only minor disability, compared to 26.5% of those without EVT [6]. This translates to a number needed to treat (NNT) of 2.6 for reducing disability [10]. It is worth noting that some patients in the control group of the EVT trials (those who did not undergo EVT) might have received thrombolysis. Unfortunately, the implementation of EVT requires specialized equipment and trained personnel, which limits its availability to larger medical centers. In cases of stroke, the urgency of the matter lies in the fact that the brain undergoes rapid deterioration shortly after the onset [11]. This time sensitivity greatly influences the effectiveness of both thrombolysis [12] and EVT treatment [13]. Thus, prompt access to EVT becomes a crucial factor. Smaller stroke centers need to respond swiftly to ensure that individuals eligible for EVT are efficiently transferred to centers equipped for the procedure. Eligibility for EVT hinges on the results of a patient’s imaging [8,14]. The regular CT images offer insights into the size of the ischemic core; only patients with a small core are considered suitable since this signifies the presence of recovered brain tissue. The CT angiogram (CTA) confirms the clot’s location within a large, reachable blood vessel which is compatible with the EVT technique. Lastly, the assessment of collateral circulation is also vital, as effective collaterals sustain brain function during transfer and the EVT intervention [15]. Despite our knowledge regarding the identification of EVT eligible patients through imaging, considerable ambiguity persists regarding the optimal approach for choosing patients to transfer from distant medical facilities where only alteplase treatment is feasible to centers equipped for EVT. A study conducted in Ontario revealed that among patients with LVO who were transferred with the intent of undergoing EVT, only 34% received the treatment. This implies a significant lack of resources, given that upon arrival, 66% of those transferred for EVT were considered ineligible for the procedure [16]. Corresponding data from the United States present a similar pattern, with only 27% of transferred patients ultimately undergoing EVT [17]. This assessment is typically conducted using multiphase CTA (mCTA) or CT perfusion (CTP). Moreover, the eligibility criteria for EVT continue to be predominantly time-dependent [12,15]. Nonetheless, various trials have demonstrated that a carefully chosen subset of patients who arrive later (even up to 24 h after onset) can still derive advantages from EVT [18,19]. This underscores the necessity for enhanced protocols to be established in the province aimed at minimizing the duration between a patient’s arrival at a peripheral facility and their subsequent transfer to EVT equipped centers; these intervals are referred to as door-in-door-out (DIDO) times [20,21]. Therefore, the efficiency of both hospital care and the transfer process in EDs plays a crucial role in determining the eligibility of patients for EVT, representing pivotal factors from both patient and clinical standpoints [22].

The deleterious effects of delayed treatment have been observed in other emergency conditions such as early thrombolysis and percutaneous coronary intervention (PCI) in AMI or heart attack [23]. Myocardial infarction (MI) is the result of a partial or complete occlusion of blood flow to a segment of the heart muscle. AMI might not exhibit noticeable symptoms and go unnoticed, or it could be demonstrated as a catastrophic occurrence causing a sudden drop in cardiovascular function and unexpected fatality. The primary cause of most AMIs is underlying coronary artery disease, which stands as the top mortality factor around the world. In cases of coronary artery blockage, the heart muscle is deprived of oxygen. AMI has conventionally been categorized into ST elevation or non-ST elevation myocardial infarction [24,25]. However, treatments are comparable between these two categories, and a comprehensive overview of the general management of AMI is provided for clarity. Despite significant advancements in prognosis over the last decade, AMI remains a prominent cause of illness and death globally [26]. This progress can be attributed to various noteworthy trends, including enhancements in risk assessment, broader adoption of an invasive approach, establishment of care systems that prioritize prompt revascularization through procedures like percutaneous coronary intervention (or fibrinolysis), developments in antiplatelet medications and anticoagulants, and increased utilization of secondary preventive measures such as statins. Extended periods of inadequate oxygen delivery to the heart muscle can result in the death and decay of myocardial cells. As the primary goal of thrombolysis is to swiftly reestablish blood circulation to the at-risk heart muscle to protect its cellular structure and operation, the factor of time becomes a critical limitation that hinders the beneficial outcomes of thrombolysis and PCI [27].

The COVID-19 pandemic and the occurrence of acute stroke and AMI may lead to increased rates of illness and mortality in various communities due to the delay in medical treatment [28,29,30,31]. This risk is particularly notable for underserved communities, which have been disproportionately impacted by the pandemic and depend more heavily on EDs to fulfill a significant part of their healthcare requirements [32,33,34].

Finally, this narrative review aims to review the application of DEA, as an important approach of MCDM, to EDs and the management of emergency conditions. Initially, we review the correlation between the notion of ‘efficiency’ in DEA and ‘convex efficiency’ in MCDM through a basic model. Through some related references, we demonstrate that certain integrated methods such as machine learning and simulation suggested in the DEA field for addressing MCDM issues contradict fundamental normative principles that are widely acknowledged. The next sections of this paper are structured as follows.

Section 2 considers techniques and views including five subsections: Section 2.1 analyses articles that apply basic DEA models for efficiency analysis in EDs; Section 2.2 examines articles that integrate basic and advanced DEA approaches in EDs; Section 2.3 considers articles that use DEA time series models in EDs; Section 2.4 reviews articles that integrate simulations, ML, and MCDM with DEA for the management of emergencies in EDs; and Section 2.5 reviews various DEA models for the acute management of stroke. Section 3 includes a discussion and limitations of this study, and finally, Section 4 provides a conclusion and suggests the focus of future studies.

## 2. Techniques and Views

This section comprises five subsections that encompass diverse integrated DEA approaches, all aimed at illustrating the role of these approaches in relation to EDs.

### 2.1. Applying Basic DEA Models for Efficiency Analysis of EDs

The two basic DEA models are the Charnes, Cooper, and Rhodes (CCR) model, which assumes constant returns to scale (CRS) [35], and the Banker, Charnes, and Cooper (BCC) model, which considers variable returns to scale (VRS) [36]. There has been constant modification of the basic DEA model over time, and the discussion about the dominance of the two basic models has continued. The suggestion of a superior model is unlikely, as each model’s performance depends on the examined data sets. In their study, Banker et al. [37] found that the CCR model delivers superior results when applied to limited sample sizes, covering up to 50 DMUs. On the other hand, the BCC performs more effectively when dealing with greater sample sizes, comprising at least 100 DMUs. The input-oriented model aims to minimize the input factors for a required output level, and output is kept at a constant level. The output-oriented model maximizes the outputs. In contrast, input is kept at a constant level. Models that focus on both inputs and output aim to maximize the outputs, minimize the inputs, and thus maximize the efficiency.

The initial study was conducted by Hao et al. [38] in 1978. They suggested the CRS approach assisted by multiple regression analysis to assess the efficiency of acute care veteran’s affairs hospitals including the hospitals’ EDs. The study excluded hospitals that were smaller in scale, housing fewer than 100 beds and those that had not been operating for 12 months. Various forms of analysis were applied to evaluate the efficiency of these hospitals. The two types of analyses used in their study were assessment of productivity and input-oriented analysis, with a focus on key outputs of medical procedures, patient releases, and visits to the emergency room and outpatient services. Therefore, the techniques employed in their study rely on quantitative inputs, including the number of staff, the number of medical beds within the hospital facility, and the corresponding number of full-time nurses and physicians. It is important to note that none of these inputs or outputs were measured in monetary units. Consequently, they were not susceptible to fluctuations in the currency’s value or changes in the dollar’s cost across different locations in the country where the hospitals were located. Multiple regression analysis was employed in combination with DEA to determine the relative efficiencies. The outcomes of the study showed that approximately half of all hospitals could improve their efficiency. Additionally, the study found that the dimensions of the hospital, inpatient surgical procedures, outpatient surgical procedures, and combined emergency room and outpatient visits were factors that had a significant impact on good efficiency values. Finally, they provided valuable insights into the efficiency of acute care veteran’s affairs hospitals and identified areas where improvements could be made. This research methodology allowed for a comprehensive evaluation of the hospitals and provided a basis for further analysis and potential enhancements in healthcare.

**Figure 1:**
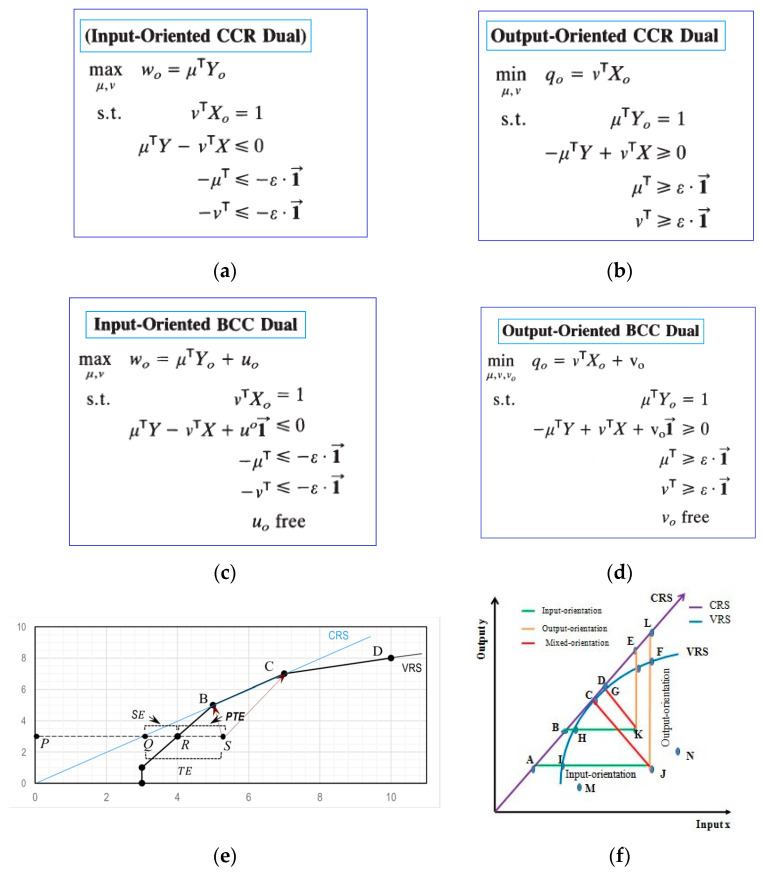
Basic DEA models: (**a**) input-oriented CCR dual model; (**b**) output-oriented CCR dual model; (**c**) input-oriented BCC dual model; (**d**) output-oriented BCC dual model; (**e**) visual representation of technical efficiency and its breakdown from various sources in a graphical format including PTE, TE, and scale SE; and (**f**) Frontier lines considering input- and output-oriented visualization [35].

Figure 1 summarizes basic DEA models including the input-oriented CCR dual model (a), output-oriented CCR dual model (b), input-oriented BCC dual model (c), and output-oriented BCC dual model. Figure 1d provides a visual representation of technical efficiency and its breakdown from various sources in a graphical format considering pure technical efficiency (PTE), technical efficiency (TE), and scale efficiency (SE), and (e) shows Frontier lines considering input- and output-oriented visualization.

Table 1 summarizes the characteristics of input- and output-oriented basic models.

Many research papers have explored basic DEA models for public hospitals and acute care centers [39,40,41,42,43]. However, in this narrative review, our focus is only on articles applying basic DEA models for EDs, which are discussed below. 

Akkan et al. [44] applied VRS and CRS models for the efficiency evaluation of EDs of seven general hospitals in Istanbul’s Beyoglu state hospitals. Four essential and interconnected variables were determined for assessing the efficiency of the EDs. These variables were carefully selected based on the critical and relevant data that were accessible. The first two variables were considered as inputs: the total bed capacity within the ED, and its corresponding level. The other two variables were regarded as outputs: the number of patients seeking emergency medical attention attended to in the ED, and the number of patients referred from the ED. Out of these variables, the classification or status of the EDs held significant importance as it influenced the selection of the appropriate DEA model and set the suggested model apart from many other models. DEA, assisted by statistical methods, was applied to make it easier for hospital managers to extract hidden rules. The small number of hospitals in their study was a weakness.

EDs play a vital role in Jordanian hospitals. Waiting times for ED patients were a critical and common problem. Therefore, Al-Refaie et al. [45] proposed a DEA-based approach to decrease the average waiting time for patients in the ED, improve the nurses’ efficiency, and increase the number of treated patients. The inputs in this scenario included the number of nurses and the typical length of stay (LOS) in the ED, where smaller values were favored. On the other hand, the outputs consisted of the average percentage of nurses and the number of patients served, where larger values were preferred. A proposal for a cellular service system was made, and it was implemented to schedule ten nurse appointments. To assess the performance measures for each design, the simulation was executed with ten replicates, each spanning one month (672 h). The optimal outcome was established by applying aggressive CRS formulation. The results showed that the optimal approach relies on distribution of workloads among different individuals or teams, which reduced patients’ mean waiting time from 195 to 183 min, increased the number of patients attended from 8853 to 8934, and improved the nurses’ operation from 52% to 62%. Ultimately, the adaptability of nurses within cellular service systems provides valuable support to hospital administrators aiming to improve the efficiency of the ED.

Chu et al. [46] applied the VRS assisted by multi-objective linear programming (MOLP) for allocation of healthcare resources in hospitals during times of public health emergencies in China. Initially, the DEA was implemented to guarantee that the ED can constantly utilize state-of-the-art knowledge through all operational periods. Each hospital was considered a DMU that utilized various inputs to create desired outputs. The suggested inputs for a DMU consisted of four main factors, including the number of doctors and nurses, the number of ICU beds, personal protective equipment (PPE) requirement, and fixed assets. The outputs comprised the number of patients admitted, the number of patients released, and the number of fatalities or the number of deceased individuals. The number of patients admitted and the number released were considered desirable outputs, representing successful medical care. Conversely, the number of fatalities was classified as an undesirable output, reflecting the negative outcome of patient care. According to the suggested model, two models for assessing efficiency were applied to determine the effectiveness levels of EDs before and after resource allocation. The findings indicated that all the EDs achieved efficiency following the allocation of medical resources, and therefore, an innovative resource allocation possibility was determined. For the MOLP, the first objective was to optimize the output, while the second objective was to link the allocated resources to the operation size of each ED.

Omrane et al. [47] proposed a VRS input-oriented model for ambulatory care departments. Four EDs demonstrated a relatively effective performance throughout the three years. Using a DEA model, they evaluated relative efficiencies by considering the available personnel (physicians, nurses, and managerial personnel) as inputs and the number of outpatients receiving treatment as the output. Consequently, they computed efficiency scores for each ambulatory care unit from 2014 to 2016, allowing them to pinpoint the inefficient units in terms of resource utilization and outpatient treatment. The findings from this research were regarded as valuable for managers in assessing the potential reductions in human resources in outpatient units without compromising their ability to fulfill tasks, and managers could thereby avoid wasting limited resources. They identified potential adjustments based on their relative efficiency to aid decision-makers in hospital management and enable the optimal allocation of human resources. These adjustments aimed to help address inefficiencies in certain EDs and implement appropriate countermeasures.

Ketabi et al. [48] suggested a CRS input-oriented model to evaluate and compare 24 EDs in Iran. The selected factors were categorized into two groups: the first subset included input factors such as the number of active beds, physicians, nurses, and medical equipment. The second subset consisted of output factors, including the number of discharges, the proportion of cases where revival or recovery occurs, typical duration of waiting, and the level of contentment or satisfaction expressed by patients. The model can be employed to identify the reasons for inefficiency and determine strategies for enhancing performance. Based on the reported data, 37% of EDs were inefficient. The primary causes were the surplus of medical devices and staff members. Therefore, DEA offered a collection of anticipated input/output quantities or levels that could make EDs comparatively effective.

During the COVID-19 pandemic, physicians and EDs faced significant challenges. The increasing numbers of patients seeking treatment in EDs led to overcrowding, subsequently impacting the quality of services provided. Consequently, the management and operation of EDs became even more pressing during the pandemic. Addressing this issue, Taghipour et al. [49] initially applied four basic input- and output-oriented DEAs to assess the efficiency of EDs located in the provinces situated in central Iran. The main factors were door-to-doctor time, number of admitted patients, employee absence rate, percentage of complaints handled, number of patients waiting in a queue, number of test kits, time required for receiving the test results, and finally the proportion of isolation rooms. These factors related to the ED are those theoretically anticipated to have an impact on the performance of EDs during the pandemic. For their research, indicators with favorable outcomes with lower levels/ranks (examples include the duration of hospitalization and the time spent in boarding) were designated as inputs. Conversely, indicators with desirable outcomes with higher levels/ranks (including the number of patients admitted and the number of test kits) were assigned as outputs. Subsequently, they conducted a sensitivity analysis to identify the key factors impacting the efficiency of this department. Their findings highlighted the significant factors influencing efficiency, which were high numbers of admitted patients, ward congestion, and the extended time taken to report COVID-19 test results.

### 2.2. Combining Advanced and Basic DEA Approaches in ED Applications

An ongoing debate in DEA concerns the choice between input-oriented and output-oriented approaches. Charnes et al. [50] proposed a third model, the additive model (ADD), which integrates both input- and output-oriented approaches. The input and output slacks are determined in ADD and optimized for the DMU to maximize their value. Thus, ADD minimizes the inputs while concurrently maximizing the outputs. Furthermore, inefficiencies extracted from slacks are provided in the analysis score. However, the initial ADD model should provide scalar efficiency evaluation.

**Figure 2:**
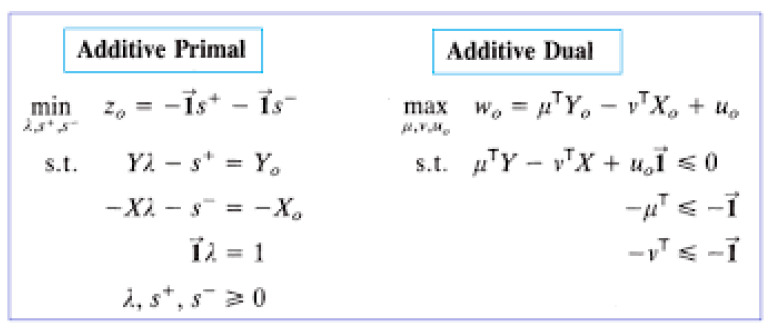
Additive DEA models: additive primal model and dual additive model.

Figure 2 summarizes the ADD models, including the additive primal model and additive dual model.

The form on the left side of Figure 2, is known as the envelopment form, representing the primal problem, whereas the form on the right side of Figure 2 is referred to as the multiplier form, representing the dual problem. Table 2 summarizes the characteristics of the ADD.

Tone’s [51] development of the slacks-based measure (SBM) suggested a new idea to address this issue. The SBM continuously reduces within each slack, with its measurement ranging between zero and one. SBM sets aside the notion of proportional adjustments in both inputs and outputs and directly addresses the concept of slacks. This method has three variations, specifically input-oriented, output-oriented, and non-oriented approaches.

The SBM models are created to fulfill the following two requirements:

**Units invariance:** The measurement remains unchanged regardless of the units used for the data.

**Monotonicity:** The measurement should consistently decrease with each slack in both input and output.

**Figure 3:**
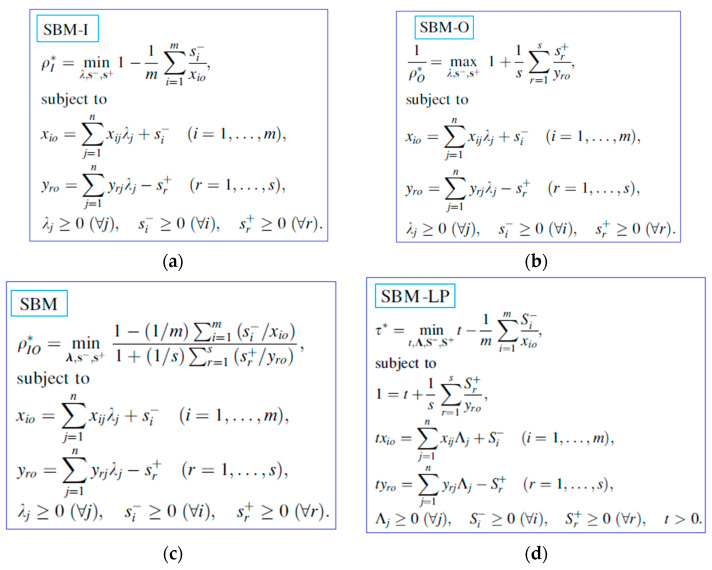
SBM DEA models: (**a**) input-oriented SBM; (**b**) output-oriented SBM; (**c**) non-oriented SBM; and (**d**) non-oriented SBM linear applying the Charnes–Cooper transformation [51].

Figure 3 summarizes the SBM model, including the input-oriented SBM model (a), output-oriented SBM model (b), non-oriented SBM model (c), and non-oriented SBM linear applying the Charles–Cooper transformation (d).

Table 3 summarizes the characteristics of SBM model.

Andersen and Petersen introduced the concept of super-efficiency in 1993 [52]. When a substantial number of DMUs achieve an efficiency score of one, super-efficiency can be employed to systematically rank all units. The idea is simple yet powerful. A DMU that has been measured is relieved from the limitations that establish the set of benchmarks or reference points. The concept of the super-efficiency model involves using standard DEA models (such as CRS or VRS) with a specific assumption: the DMU being assessed is not included in the reference set. When considering inputs, this model provides an estimate of how much a DMU’s inputs can be increased while maintaining its “efficient” position about the frontier defined by the other DMUs that are still included in the analysis. The super-efficiency score can alternatively be regarded as an indicator of stability. In other words, if the input data are susceptible to errors or undergo changes over time, the super-efficiency score allows us to assess how much these variations can occur without compromising the DMU’s efficient status.

Du et al. [53] applied advanced DEA to compute the efficiency of hospitals. They employed an integrated SBM super-efficiency model to assess hospitals providing comprehensive acute care services. The model considered the common selection of variables for inputs and outputs and considered the health outcome quality measure represented by the survival rate among the suggested outputs. In the study, every hospital within the sample was considered a DMU, which employed inputs encompassing both the physical and monetary aspects to generate outputs consisting of healthcare services and the subsequent health conditions. As a result, the proposed model evaluated both the quantity and quality of the outputs. By using this DEA model, inefficiencies in the DMUs were identified and addressed without compromising the quality of care provided.

Dexter et al. [54] performed research in which they employed data resampling methods to explore different statistical choices for super-efficient DEA. This was performed either for comparative purposes or as a reference for administrators overseeing DMUs in rural, teaching, and state hospitals. The output in this context was the number of hospital discharges, which included the specified procedures. On the other hand, the inputs comprised the number of staffed acute and intensive care beds available at the hospital, as well as the number of surgeons who carried out a minimum of three cases for any of the eight procedures, such as cardiac and neurological surgery, at the hospital. Their primary focus was on identifying the disparities in the outputs, considering slacks adjusted to account for favorable inclination or tendency, to determine areas where improvements could be made. Through numerical experiments, they observed that the estimations of the output differences did not tend towards uniformity as the values increased indefinitely, a characteristic that is commonly anticipated in standard numerical assumptions. They found that larger sample sizes did not consistently lead to more precise predictions of the output gaps. The gaps obtained from the baseline DEA correlated with the jackknife mode and the resampling mode, with or without additional data from any specific population subset. In most cases, the baseline DEA gaps were also equal to the median. These findings highlighted the importance of considering the DEA results together with a sensitivity analysis, in which one benchmark DMU is ignored at a time. The sensitivity analysis was found to enhance the effectiveness of decision support provided by the DEA by identifying the capability of a DMU to improve one or multiple outputs.

In the following, we discuss papers that specifically examine advanced DEAs in the context of EDs.

Fiallos et al. [55] suggested an SBM-VRS model to evaluate the performance of EDs. Within the EDs, there was a lack of motivation to adopt a methodology that examined the correlation between in increasing inputs and the effects on outputs and this led them to focus on models that allowed for VRS. In the model, quantity measures were utilized as inputs, while quality measures served as outputs. Their study focused on three inputs: the mean duration of individual appointments, the mean count of laboratory tests, and the mean count of radiology orders for each appointment. The only output considered was the level of non-return individual appointments within 72 h. They examined the significance of employing a sophisticated approach that recognizes the diversity of patients and ED physician encounters and the crucial role they serve as advisors for instructing other physicians. Therefore, patients were grouped according to their representative medical problem, and ED physicians evaluated each group independently. Performance differences were evident among physicians in each complaint group and set. A secondary categorization separated patients according to whether they were attended by a trainee in addition to the primary joining physician. Practically every ED physician exhibited improved performance when they were not assisted by beginners.

Agovino et al. applied an SBM to EDs in Italy [56]. The main objective of the study was to clarify changing aspects of the efficiency of the ED during an emergency medical assistance program. Thus, they analyzed the city of Sorrento in two stages. An efficiency analysis was conducted using two inputs and two outputs. The outputs were categorized either as favorable results, represented by the number of individuals seeking medical attention or treatment who experienced improved medical conditions or ailments related to a person’s well-being after medical care provided in the ED or as unfavorable outputs, represented by the number of individuals who experienced worsening health conditions after medical treatment. The main goal was to assess the effectiveness of first aid in enhancing patients’ health conditions. Both output variables were established based on the triage code, which serves as a reliable indicator or factor that can help determine a patient’s degree of immediacy and, consequently, the ED’s ability to provide appropriate treatment. Additionally, the check-out code was also considered in the analysis. They incorporated two time-based variables as inputs for their analysis: access time (AT), and healthcare time (HT). Both factors were computed as the daily wait time median. The AT was used to obtain the period starting from the time a patient entered the phase of triage or the triage level till a physician conducted the first examination. This quantity represented the duration of time spent waiting to receive first aid and critical information provided for patient healthcare support. A greater value of AT indicated poorer performance of the ED. On the other hand, HT quantity represented the duration of health treatment from the patient’s initial health examination until their discharge. First, they used an SBM to explore the changing efficiency patterns of the Sorrento ED. Secondly, they utilized a filter to verify if variations in ED efficiency were implemented through the gradient of the supply curve for first-aid services. The researchers concluded that a potential solution might involve adopting a flexible management approach for medical and nursing staff. This would involve incorporating daily and seasonal planning that considers fluctuations in work intensity. Such planning would empower Sorrento’s ED to function more efficiently, even during periods of reduced demand.

Since health transformation programs (HTP) and EDs are the most critical aspects of the health sector, Bozdemir et al. [57] applied super-efficiency to VRS and CRS models to remove multiple efficient DMUs from EDs. To assess the efficiency of health activities, three inputs and three outputs were considered. The inputs comprised the gross domestic product (GDP) share of current expenditure on health, the number of graduates from medical faculties per 100,000 population, and the rate of deaths per 1000 live births (infant mortality). On the other hand, the outputs consisted of the number of beds per 1000 population, life expectancy (representing the regular life expectancy at birth, assuming mortality rates remain constant across different age groups), and the percentage of the total population aged 65 and over. The main objective was to evaluate the achievement, effectiveness, and endurance or long-term viability of HTP; in this situation, they considered two separate case studies. First, Turkey’s efficiency in the health sector was assessed during 2003–2016, and based on this first case study, Turkey was inefficient during 2003–2012. The main reason for their being inefficient was a lack of investment. The second case study compared Turkey with nine other countries based on GDP, and it was observed to be efficient except in 2007. Finally, Turkey ranked fifth among the ten countries regarding the average efficiency score.

Ngee-Wen et al. [58] utilized a retrospective observational approach, analyzing data on support staff, doctors, discharges, arrival times to consultants, and LOS. For their study, two inputs were selected: full-time equivalent staff (representing the total number of support staff, including nurses and medical assistants) and full-time equivalent medical staff (comprising the total number of doctors, including medical officers, specialists, and consultants). Moreover, one output was considered, which was the number of discharges (representing the total number of discharges in the ED for a given year). These inputs and outputs were selected based on their extensive use as variables in DEA healthcare research. Two additional outputs were included: time from arrival to consultation (representing the total number of patients with a time from arrival to consultation of less than 90 min) and LOS (representing the total number of patients with an LOS less than 120 min). The inclusion of these outputs was determined by lean key performance indicators employed to track the results of lean healthcare implementation. Efficiency scores were then computed for 20 public EDs in Malaysia using SBM. These scores were then compared before and after the implementation of lean practices. In evaluating the outcomes of lean healthcare, several key performance metrics were typically employed. However, the current deficiency lies in the absence of adequate tools to assess efficiency in this context. After the lean healthcare implementation, 13 out of the 20 EDs showed progress in reducing the LOS and the time it takes for a patient to arrive and see a consultant or specialist. On the other hand, it is worth noting that out of these 13 public EDs, only 9 of them experienced an improvement in their efficiency score. Lean healthcare has been proven to positively impact the efficiency of specific EDs. The SBM model provided comparative analysis capabilities and valuable information for slack removal, which can complement the principles of continuous improvement in the context of lean practices.

Azadeh et al. [59] assessed three categories of inaccuracies, which involved insecure transport, multiple or recurrent needle punctures into a vein, and errors in the process of collecting samples. The suggested inaccuracies were incorporated into a simulation model. A total of seventy suitable settings, validated by specialists in ED, were outlined to evaluate different options. These settings were then analyzed and assessed using stochastic DEA (SDEA) to identify the optimal solutions. In their study, the inputs consisted of cost, number of nurses, and number of physicians, while the outputs included waiting line, the length of time a patient spends or stays in a particular situation or medical setting, and the number of three distinct mistakes that were made or committed. The findings showed that adding additional nurses and physicians or incorporating a larger number of nurses and physicians in the ED would lead to a reduction in human errors, patient duration, and queue length.

### 2.3. Applying DEA Time Series Models to EDs

The Malmquist productivity index (MPI) and window analysis are the two most common DEA time series approaches for measuring productivity and efficiency, respectively. Various methods can be employed in productivity evaluations to calculate productivity changes, including the Fisher, Tornqvist, and Malmquist indices. Among these, the Malmquist total factor productivity (TFP) index, introduced by Malmquist [60], is the most used analytical tool for assessing productivity changes.

The MPI offers three key advantages over the Fischer and Tornqvist indices. Firstly, there is no need for information on optimizing earnings or minimizing expenses. Secondly, there is no need for data on input and output prices. Lastly, when applied to panel data, the MPI enables the separation of productivity changes into two separate elements: technical efficiency (also known as catching up) and technical change (which refers to alterations in best practices).

The DEA window analysis operates based on the concept of moving averages, as initially proposed by Charnes et al. [61], and it proves valuable in identifying efficiency changes observed in a unit over a period. In this approach, each unit is considered a separate entity in separate periods. Consequently, the efficiency of a specific component in a particular period is evaluated not only by its efficiency during different timeframes or in alternate periods but also by the efficiency of other components. By incorporating additional data points into the analysis, this method becomes particularly useful for situations with small sample sizes.

Trakakis et al. [62] conducted a study using the input oriented MPI approach, considering both VRS and CRS, to examine the total productivity of 155 rural primary care hospitals in Greece. Twelve outputs were identified, which included the total number of nursing activities, small-scale surgical procedures or minor surgeries, dental treatments, cases of long-term or persistent medical conditions, emergencies, urgent events, transcriptions, bio pathological and laboratory exams, vaccinations, and vaccinations for children and teenagers. Inputs consisted of the total number of managerial employees, doctors, nursing staff, and non-medical staff. The research aimed to assess the productivity of each of the 155 DMUs in Greece and analyze how it changed during the period from 2016 to 2018. Additionally, the study evaluated the overall productivity change of all 155 DMUs over time. The mean value analysis revealed there was a decline of 0.9% in overall productivity between 2016 and 2017 and a further reduction of 5.2% from 2017 to 2018, resulting in a total reduction of 3.1% in the productivity of all 155 DMUs. The findings from this model can provide valuable insights into the performance of each rural health clinic. In a related study, Bağcı et al. [63] proposed a time series DEA-based MPI using CRS and VRS models from 2011 to 2016 in rural hospitals in Turkey. As input variables, the study considered the total number of beds, specialists, residents, general practitioners, nurses, and midwives; other medical personnel wages and benefits; other service expenditures; raw materials and supply costs; and overall administrative costs. As for the outputs, the study considered the number of inpatients, outpatients, and surgical operations for three suggested groups; and working capital turnover. The research revealed that the proportion of efficient rural hospitals decreased from 2011 to 2016, indicating that the organizational and financial management of rural hospital supervisors may have contributed to lower productivity.

Zhou et al. [64] applied the same MPI time series DEA, assisted by the Tobit statistic model, to analyze factors influencing productivity in 28 urban and rural areas. The input variables in the study consisted of the number of institutions, beds, and health technicians. Alternatively, the outputs involved the numbers of outpatients and emergency visits, as well as the number of discharged patients. They found that the concentration or distribution of the population and the ratio of dependents (non-working population) to the working-age population were the primary aspects influencing the technical efficiency of rural areas. To improve productivity, the authors recommended improving medical technology in rural clinics through technology restoration.

Jia et al. [65] performed a time series DEA window analysis, assisted by CRS and VRS, to assess the operational efficiencies of EDs in five hospitals over seven years. The data pool used in the study was provided by health authorities. Consequently, they considered two inputs, including the real count of beds and staff members at the termination of the period. Three outputs were considered: the number of outpatients and EDs, the number of patients discharged, and the mean duration of hospital stay at the termination of the period. The results revealed that the sample EDs exhibited an overall increasing trend in their operational efficiencies, comprising TE, PTE, and SE, over seven years. However, there was a brief decline shortly after the hospital’s reorganization, with PTE showing more improvement in comparison with SE. Particularly, the creation of separated hospitals did not have a lasting adverse impact on efficiencies in hospital operations. The final findings suggested the impact of increasing SE through enhanced organizational management and highlighted the benefits of adopting different branch organizations, merging, and restructuring. The study provided valuable insights into the real-world operation of DEA window analysis for measuring basic operational efficiencies of EDs.

Mohd Hassan et al. [66] proposed an evaluation of cross-sectional efficiency for 76 EDs. For this analysis, the cost of ambulance services was considered as the input, and the output variables included the extent of geographical coverage or distance span (kilometers), the number of transferred patients, and utilization hours. The evaluation of TE assumes CRS is known as well as the overall technical efficiency (OTE). When VRS is considered, OTE can be divided into two separate components: PTE or managerial efficiency, and SE. PTE and SE are distinct from each other and cannot be merged or combined. In the study, further assessments were applied to examine the OTE, PTE, SE, and RTS for different health facilities and geographical regions. For these analyses, the Mann–Whitney U-test and chi-square test were applied. The primary reason for the disparity in OTE among hospitals and EDs was their operating size rather than the PTE.

### 2.4. Integrating Simulations, ML, and MCDM with DEA for the Management of Emergency Conditions in EDs

DEAs can be integrated by various commonly used techniques, such as MCDM, artificial neural networks (ANN), logistic regression, and discrete event simulation (DES).

MCDM originated from operations research (OR) and includes various approaches. MCDM is a method for ranking a finite number of alternatives assisted by multiple criteria. It evaluates and selects alternatives that fit the objectives and requirements [67].

DEA benefits from the incorporation of ML algorithms, such as ANN and logistic regression. ANNs draw their inspiration from the brain’s primitive sensory treatment models, which can be simulated using a network of model neurons in a computer. By implementing algorithms that mimic real neuron processes, the network can “learn” and effectively solve various problems. The ANN collects inputs from different units and produces an output of one if the total input exceeds a specified threshold, otherwise, the output is zero. The output transitions from 0 to 1 when the total weighted sum of inputs reaches the threshold [68]. In addition, logistic regression is a widely utilized ML algorithm, specifically categorized as a supervised learning technique. It is employed for predicting categorical dependent variables based on a given set of independent variables. Logistic regression is well suited for forecasting the outcome of a categorical dependent variable [69].

Simulation studies have long been applied in healthcare to address delays and challenges associated with the healthcare system. Researchers have explored numerous alternatives, considering factors such as processes within the organization and level of staffing, and applying simulation models to enhance the performance of EDs and decrease patient waiting times. DES, which was proposed by Günal et al. [70], is a prominent tool employed for analyzing and optimizing healthcare systems. It is frequently integrated with DEA in various research articles. DES evaluates the functioning of a system as a series of distinct events occurring chronologically. Every occurrence takes place at a designated moment in time and results in a state of systematic modification. When there are no changes between consecutive events, the simulation time can instantly advance to the time of the next event, known as the next-event time progression.

Several studies have proposed integrating DEA with ANN or logistic regression for acute care hospitals [71,72,73,74]. Additionally, many research papers have suggested integrating DEA with DES for acute care centers [75,76,77,78,79]. However, in this narrative review, our primary focus below is only on the evaluation of articles that consider integrating the suggested approaches with DEA models in EDs.

Despite the critical role of ED performance measurement, commonly applied metrics need to be normalized. The objective of the research paper by Kang et al. [80] was to suggest an efficiency indicator that supports evaluating EDs in connection with TE and SE. They explored critical exogenic components concerning the TE of EDs. According to the provided information, the research was an initial study analyzing the scale and technological efficiencies of EDs. Their study applied input-oriented DEA models that considered six inputs and outputs and created an efficiency ranking for specific EDs. The inputs for the evaluation consisted of three variables: the number of ED beds, clinical staffing working hours, and non-clinical staffing working hours in the ED. On the other hand, the outputs included three measures: the daily number of patient visits, the average LOS, and the rate of leaving without being treated. The DEA analysis suggested that a considerable number of EDs might not require adjustments in their operation range to increase efficiency. Alternatively, they might have to re-engineer specific procedures to apply the available inputs effectively. The logistic regression supported the finding that different operational features within the ED, including the LOS, and the percentage of patients arriving by ambulance, were related to the TE of EDs. Using these models as strong tools for comparing and evaluating performance, the results can serve as a foundation for improving ED performance by focusing on critical hospital resources.

EDs need to adopt effective systems that reduce expenses while ensuring satisfactory levels of care. The main objective of Weng et al. [81] was to create and implement a combined approach using DES and DEA. Their study considered different types of ED resources as inputs, including the numbers of physicians, nurses, and beds. The output aimed to evaluate how modifications in these input levels influenced the efficiency of ED operations, leading to the identification of the most efficient resource allocations. This approach aims to assess possible bottlenecks, optimize throughput, and find solutions to decrease patient waiting times in the ED while enhancing patient satisfaction. The same integrated approach was applied by Aminuddin et al. [82] to determine the highest potential demand that the ED can handle using its existing resources. DES was employed to examine the waiting time patterns of ED visits and forecast the peak demand. DES-DEA was applied to identify the optimal decisions concerning the number of resources (doctors and nurses) necessary to sustain their efficient services. The inputs in this approach include the number of physicians, nurses, and the overall mean waiting time for patients, and the outputs consist of the average utilization of doctors, the average utilization of nurses, and the number of patients served. The primary goal was to minimize the overall mean waiting time while achieving the lowest possible utilization of resources, considering the average provided rate of resource utilization and the number of patients attended. The most effective improvement was identified through the implementation of the BCC input-oriented method and super-efficiency method. The proposed improvement can serve as an initial benchmark for hospital administration to make informed decisions while addressing the problem of overcrowding.

Due to demographic change and growth in the number of aging people, timely access to health services has become increasingly difficult. This situation creates many difficulties for patients and medical facilities. Acute hospitals are experiencing an unprecedented level of overcrowding because there is a shortage of available acute beds. Consequently, patients in need of treatment experience prolonged waiting periods as healthcare providers focus on whether to admit them, transfer them to another facility, or discharge them to go home. These extended waiting periods frequently lead to patients entering various locations within the hospital, resulting in risks to patient safety and reducing the level of service provided but raising the expenses associated with medical care.

Keshtkar et al. [83] proposed an integrated simulation methodology that allowed hospital managers to consider the patient waiting challenge. Merging dynamics and DES assisted the manager in facilitating the difficult patient movement at both larger, overall levels and smaller, specific stages. Design of experiment (DOE) and DEA were incorporated into the simulation to efficiently evaluate the operational consequences of different management interventions. In CRS and VRS models, some DMUs are efficient with an efficiency score of one, and some DMUs with an efficiency score of lower than one are inefficient. Sometimes, several DMUs may receive an efficiency score of one. Thus, to address this issue, the super-efficiency method was applied by ordering DMUs based on their efficiency levels. The DMU with the highest super-efficiency score is considered the best. The VRS output-oriented model performed better than other models and was considered for ranking.

The gap between the number of doctors and nurses and the patient ratio creates a bottleneck in the available resources, leading to long waiting times for patients, particularly after office hours, and during weekends. To achieve the optimal resource allocation for the two shift groups, a combination of DES integrated with BCC input-oriented and super-efficiency methods was proposed by Yusoff et al. [84]. This approach generated multiple resource allocation options for doctors and nurses, amounting to 64 options available for weekdays and 729 options available for weekends. The DES model provided the values for the mean waiting time, the mean operation rate of physicians, the mean operation rate of nurses, and the number of patients who have been attended to or treated. In terms of preference, the number of physicians, the number of nurses, and the mean waiting duration were considered inputs, since values that are smaller in size were favored. Conversely, the mean usage or occupancy of physicians, the mean usage or occupancy of nurses, and the number of patients attended to or treated were designated as outputs, since higher values were favored for these variables. The findings revealed that the optimal distribution of physicians and nurses during weekdays consists of a team of three physicians and three nurses for each work period. During weekends, the most effective group consists of four physicians and four nurses for each work period. These recommended arrangements have resulted in reduced average waiting times, improved utilization of medical staff, and an increased number of patients attending during weekdays and weekends. The same DES-DEA was proposed in recent studies [85,86]. The main objective was to increase the efficiency of hospital EDs, aiming to minimize patient waiting times and optimize resource utilization. The outcomes underscored the significance of maintaining a well-balanced number of doctors within the ED to maintain an acceptable patient throughput time. Finally, in the last suggested article examining DES, Rabbani et al. [87] proposed a DES-DEA approach. Their paper analyzed the clinical pathway as a crucial element in the integrated simulation of the ED due to the significant interactions of laboratories, radiology departments, and pharmacies. EDs deal with a range of patients, each having unique priorities. This leads to the necessity for distinct response variables, creating a multi-response optimization problem. To address these resource allocation challenges, a novel approach that combines DEA, DOE, multi-layer perceptron, ANN, and radial basis functions was introduced. Due to the expensive and limited nature of healthcare resources, the model incorporated budgetary and resource restrictions.

The healthcare sector is facing a notable and continuously expanding issue with human error. Yazdanparast et al. [88] suggested an analysis of both resource allocation and human error to optimize the use of resources in an ED. The six inputs were the number of triage nurses, physicians, nurses, beds, CPR units, and oxygen capsules, while the six outputs were the average wait time of patients, considering the weight or significance of each case; the average wait time for patients during the triage process; the rate or occurrence of errors related to skills or competencies; a score or measure of redundancy or duplications; the average waiting time for beds or the typical duration patients wait to be assigned a bed; and fee. The algorithm consists of four main components: simulation, ANN, DOE, and fuzzy DEA (FDEA). The approach aimed to optimize multiple aspects, including human error, cost, wait time, patient safety, and productivity. The simulation helped establish the link between human resource utilization and human error. Furthermore, ANN was employed to predict response variables, while FDEA was applied to determine the optimal scenario.

Kang et al. [89] proposed a data-oriented framework to compare and establish efficient EDs, along with implementing their most effective methodologies. Initially, they employed DEA to recognize the frontiers of efficient operations in EDs. Then the ED benchmarking alliance database from 2012 categorized 449 EDs into six groups and assessed the efficiency of each ED within those groups. The components considered as inputs in this context were the number of ED beds, clinical staffing working hours, and non-clinical staffing working hours within the ED. On the other hand, the outputs consisted of the daily number of patient visits, the average LOS, and the rate of patients leaving without being treated. After obtaining the efficiency rankings, logistic regression was employed to determine the specific attributes of EDs that influenced their classification as either efficient frontiers or inefficient units. The findings revealed that the efficiency of the EDs was significantly influenced by the proportion of admitted patients entering through the ED, the utilization of a mid-level provider intake model, the presence of a fast-track area, and the overall patient volume.

Labijak-Kowalska et al. [90] conducted an extensive investigation into the resilience of efficiency results concerning various input and output weights. They achieved this by applying mathematical programming and the Monte Carlo simulation. They examined three inputs, namely, the mean duration of each patient’s appointment, the mean number of laboratory tests conducted during each patient’s appointment, and the mean number of radiology requests made during each appointment. Additionally, the output used was the level of non-return individual appointments during a time frame of 72 h. They concentrated on the subset of patients who primarily presented expressions of discomfort in the abdominal region and constipation. However, during their analysis involving multiple scenarios, they also considered two additional groups with complaints connected to instances of fever, as well as injuries affecting either the lower or upper limbs, head, and wounds involving lacerations or punctures. Specifically, they employed the ADD model for their analysis. They concentrated on evaluating physicians’ performance in handling different groups of patients’ complaints. The results that were obtained highlight how much physicians’ performances depend on the specific weight vectors that were selected. Additionally, they provided a foundation for creating a plan to enhance the performance of physicians who are not meeting performance expectations or are performing below the desired standard. The findings also assisted in deciding on the main priorities for a practice-oriented model and detecting the most challenging issues raised by patients.

DEA assisted by MCDM has been proposed in analyzing EDs [91,92]. Abdel-Basset [93] applied DEA integrated with MCDM to assess the efficiency of EDs in 20 hospitals. This assessment was based on two key criteria: the number of patients who received treatment; and the impact on the standard of living experienced by a patient, which was evaluated by applying 11 different factors. In this research, 11 inputs that directly and indirectly impact the operations of the ED were taken into consideration. The suggested inputs have been verified by specialists and corroborated by prior research. The inputs included the number of unoccupied beds, the level of importance or severity of the department, the number of skilled and capable nurses, the choice of task allocation based on the patient’s medical condition or pathology, the LOS, the number of ambulances leaving or departing from a location, the process of admitting a patient to a hospital for medical care and treatment, the rate at which patients are moved or transferred to different hospital units or facilities after being admitted, the time taken for ambulance personnel to transfer a patient from the ambulance to the hospital or medical facility, the presence or accessibility of medical apparatus or devices for use, and the number of proficient or capable physicians. The study concentrated on two specific outcomes: the impact on the patient’s quality of life and the number of patients who received medical care or treatment. Using the analytic hierarchy process (AHP) as one of the MCDM techniques, the study measured the weighting of efficiency factors to achieve more precise aggregation outcomes, considering the level of contradiction between criteria values. The findings indicated that half of the hospitals (ten out of twenty) demonstrated efficient service in their EDs, whereas the remaining ten hospitals showed lower levels of efficiency.

Gharahighehi et al. [94] proposed a methodology to improve the performance of a hospital ED in Iran. The ED faced challenges due to extended waiting times and uneven resource allocation, causing issues for both patients and ED staff. To address this, the method involved simulating the patient flow within the ED, assisted by DEA-DES, to identify the bottlenecks responsible for the inefficiencies in the ED’s performance. The simulation model considered non-homogeneous patient arrivals and provided detailed representations of diagnostic procedures and medical conditions through the study and examination of bodily samples or specimens, test center testing, a medical imaging technique that uses high-frequency sound waves to create visual representations of internal body structures in a non-invasive manner, magnetic resonance imaging (MRI), CT scanning and radiology. DEA software was applied to analyze ten different scenarios, each consisting of one input and three outputs. In this context, each scenario represented a DMU. The input was the average number of patients arriving per day. The initial output was the number of left without being seen (LWOS) patients. The second output was the average waiting time, and the third was the cost. To evaluate efficiency, all the outputs were normalized using an output-oriented approach. Among the ten scenarios, four were considered efficient, achieving a maximum efficiency score of one. However, the remaining six scenarios were deemed inefficient and required adjustments or replacements. Four well-known MCDM techniques including DEA, AHP, VIKOR, and the Delphi method were applied in the study. DEA was employed to identify efficient scenarios, while AHP was used to assign weights to every individual principle. The Delphi method was applied to determine appropriate rates of usage for different resources. Additionally, the prolonged VIKOR method was used to assess and rank data based on 95% confidence intervals, which were obtained from efficient scenarios based on divergent factors. Finally, by applying the highest-ranking setting, which did not require any additional investments, the overall waiting time for acute patients could be reduced by approximately 5%.

### 2.5. Applying Various DEA Models for the Management of Stroke Emergency Conditions

DEA has been used to analyze stroke treatment in some studies. The initial study was published by Ozcan et al. in 1998 [95]. They applied CRS input-oriented DEA to analyze relations among contributors’ knowledge and technical efficiency. The four inputs considered in their study were average LOS, charges for occupational therapy (OT) and physical therapy (PT), as well as total charges. All three variables served as measures of resource usage. Health administrators, who were refunded based on the diagnosis related group (DRG) system, had strong incentives to reduce the average LOS while maintaining quality outcomes. Therefore, a lower LOS indicated higher productivity under similar circumstances. To analyze the outputs, patients were divided into two categories to consider different patient case mixes: mild and severe. Severe strokes were identified by either a coma diagnosis or having at least four diagnoses with at least one surgery performed to treat the principal diagnosis directly. The number of secondary diagnoses has been verified as an indicator of severity in studies of other medical procedures. All cases that did not meet these criteria were classified as mild stroke cases. The charge variables represented the resources required for stroke treatment. Although using true costs would have been a more accurate measure of resource usage, the necessary data was not available. As a result, in the analysis, it was assumed that charges reflected the level of resource utilization. The article examined the average LOS and the expenses related to physical therapy for cases of stroke, which were classified as mild or severe, across 214 hospitals categorized by their level of experience in stroke treatment. These factors were used as input variables in the study. The study found that, on average, technical efficiency develops with experience. Conversely, although more qualified suppliers were considered more technically efficient on average, they tended to charge more. The study further suggested that the gap in the cases of acuteness among efficient and inefficient contributors expands as the experience level increases. The reported outcomes indicate a significant ability for workers who are not operating efficiently to change their methods of practice to those of related effective workers and thereby decrease collective costs significantly.

Behr et al. [96] investigated health system efficiency at the country level based on Organization for Economic Co-operation and Development (OECD) health data. The 30-day mortality after admission to a hospital for ischemic stroke per 100 patients (based on admission data) and 30-day mortality after admission to a hospital for acute myocardial infarction (AMI) per 100 patients (based on admission data) were two specific outputs. Due to issues related to data availability and missing information, they were unable to include all aspects of the ideal typical inputs. Instead, the inputs used in the study were categorized into three broader categories. The first category was basic medical inputs, which consisted of variables such as the number of hospital beds for every 1000 individuals, the number of committed physicians for every 1000 individuals, and the number of committed nurses for every 1000 individuals. The second category was intermediate medical inputs, which included variables such as surgical procedures for cataracts, overall number of procedures for every 1000 individuals, surgical procedures for coronary artery bypass (the number of hospitalizations for every 1000 individuals), and kidney transplants (the overall number of medical procedures for every 1000 individuals). The third category was financial inputs, which involved healthcare spending as a percentage of the GDP. They categorized the output indicators into two groups: designated and non-designated. Although they acknowledged the suggested categorization was not entirely precise, they considered designated outputs to be more directly associated with the impacts of the health system compared with the undesignated outputs. The designated outputs comprised the number of infant deaths per 1000 live births, the rate of mortality within 30 days after hospital admission for ischemic stroke as the number of deaths per 100 patients using admission data, and the rate of mortality within 30 days after hospital admission for AMI as the number of deaths per 100 patients using admission data. On the other hand, the non-designated output referred to life expectancy at birth. They emphasized numerous aspects of healthcare approaches to detect inefficiencies. Perfect evaluations of health systems, disregarding limitations on data access or data limitations, and the underlying theory or theoretical foundation for a discussion under real data limitations were among the highlighted issues. This discussion highlighted prospective policy measures that could improve efficiency. Their analysis included hospitals in 34 countries assisted by the VRS model. Productivity measures relative to average DEA prices were computed. Due to data restrictions, they emphasized several aspects of each system rather than conducting a comprehensive analysis of each healthcare structure.

Another study published by Amiri [97] assessed the quality of nursing care. A cross-national study was conducted to analyze the responsibilities of recently graduated nurses in providing high-quality nursing care and optimizing patient outcomes. The article utilized the VRS model assisted by the statistical technique of a generalized linear model to investigate stroke care services in Finland. Data were gathered from 33 OECD countries, including the number of nursing graduates for every 100,000 people plus three OECD health care quality indicators (HCQI) within the acute care centers. These HCQIs included the mortality rates within 30 days, both in-hospital and out-of-hospital, for every 100 patients incorporating AMI, hemorrhagic stroke, and ischemic stroke. Additionally, four control variables were included in the analysis: the number of individuals who have completed their medical education and training, the number of nurses currently working in the profession, the distribution of doctors for every 1000 individuals (acting as substitutes or representatives for other various healthcare occupations), and the number of CT scanners per one million individuals (an indicator representing the level of healthcare expertise). Increased personnel or a larger workforce of newly qualified nurses was related to improved patient results in acute care, while the clinical efficiency of nursing graduates (which was linked to their educational level) was the critical reason for increasing the quality of DMUs and patient survival rates. Furthermore, integrating the DEA model with other linear or nonlinear programming approaches is important for a DMU’s evaluation. Conversely, one of the most difficult tasks in allocating resources within musculoskeletal rehabilitation units is providing treatment for patients with brain injuries, particularly those who have suffered a stroke or are in the post-stroke phase.

Koltai et al. [98] proposed output oriented SBM for assessing inpatient rehabilitation centers in Hungary. The four selected inputs in their study were the number of hospital beds (this refers to the overall bed capacity in recovery units, which establishes the main ability of these units to deliver medical care provided to patients with musculoskeletal conditions), the number of medical doctors (this represents the full-time equivalent (FTE)), the number of doctors employed at the rehabilitation units, the number of nurses (this indicates the full-time equivalent number of nurses working within the rehabilitation area), and the number of medical practitioners with specialized expertise (this refers to the full-time equivalent number of various specialized healthcare professionals such as verbal communication therapeutic services, mental health professionals, certified massage therapists, instructors specialized in acceptable education, physical therapists, physical therapists assistants, professionals in the field of healthcare who assist individuals in acquiring or restoring the abilities necessary for their daily activities and tasks, healthcare professionals who focus on the management and care of orthopedic devices and equipment, including braces, casts, and prosthetics, professionals who provide support and assistance to individuals, healthcare professionals who specialize in providing physical education and exercise programs tailored to individuals with medical conditions or special health needs, educators who work with students with special needs, healthcare professionals who specialize in nutrition and dietary advice, and other professionals who provide various forms of therapeutic treatment). In addition to using operating expenses and investment factors, the researchers also attempted to consider the financial aspect. However, obtaining accurate and comparable data for these financial inputs is a big challenge. The primary mode of financing for hospital units relies on the number of beds. Therefore, the number of beds was applied as an approximation for the impact of financial inputs. The four outputs included the number of patients (this represents the number of musculoskeletal patients who are discharged from the department when either their therapeutic or recovery-oriented care is completed or due to transfer to other healthcare units), a typical alteration in health condition (this indicates the typical variance in the Barthel index (BI) score at the time of admission compared with the score when leaving the hospital and reflects the change in health status during the rehabilitation period), the number of individuals with stroke and brain injuries (this value indicates the proportion of the population with complex cases among all patients), and the hypothetical potential for patients to improve their health status. This represents the average variation among the highest scores on the BI upon admission. The value is obtained by subtracting the BI from 100 and indicates the possible enhancement in the health condition that individuals can experience throughout their rehabilitation journey. As a result, the research focused on those rehabilitation centers that concentrate on patients recovering from stroke or other brain damage. The main result was the patients’ health condition in relation to change in the efficiency scores. Assessing the impact of hospital services on health improvement often proves challenging to quantify. Still, this determination is needed to connect the availability of DEA studies with the needs of decision-makers seeking DEA results.

Finally, despite the potential advantages of using operation research (OR) methodologies in the healthcare industry, there is a notable lack of research regarding the application of OR tools like MOLP in addressing complex healthcare challenges, particularly in the assessment of stroke care services. Moreover, although the existing literature emphasizes the benefits of providing appropriate care services to stroke patients, it falls short in demonstrating over an extended period the advantages of enhancing the immediate availability of different stroke care interventions for patients throughout their lifetimes. Consequently, a recent study proposed by Mirmozaffari et al. [99] aimed to address this significant research gap by filling it with valuable insights and information. They suggested a novel integrated approach to stroke care services. DEA and MOLP are extensively utilized for evaluating efficiency. Even with their similarities and overlapping principles, they have developed independently. The generalized DEA (GDEA) cannot consider decision-makers’ (DM’s) individual choices and past efficiency records. On the other hand, MOLP can include the DM’s preferences when making decisions. To address this limitation, they transformed the GDEA to MOLP using the maximum-ranked option, leading to several advantages, including interactive problem-solving, integration of the step method (STEM) to reflect the DM’s choices, elimination of the need for pre-established preference information, and application of the most preferred solution (MPS) to determine the most effective method or strategy. Their paper has the potential to serve as a starting point for various research directions. One such area involves expanding the practical use of the non-radial or non-oriented GDEA model. Additionally, an interesting subject for investigation is the exploration of robust inverse GDEA and a comprehensive framework based on the interactive GDEA dual model and MOLP. However, it is worth noting that the data used for conducting a GDEA might be imprecise. Therefore, it becomes essential to consider the concept of imprecise interactive GDEA.

## 3. Discussion and Limitations

Despite significant efforts to enhance the performance of EDs, there remains a need to further develop cutting-edge solutions for implementation within the emergency care environment. Accompanied by timely external measures, efficient emergency care networks must be established in real-world scenarios. These strategies could also be adapted and applied to support ED operations beyond the pandemic period. For instance, the integration of DEA, machine learning, and simulation techniques, which are currently used to predict health outcomes for ED patients, could also be utilized to anticipate potential health issues in individuals suffering from acute ischemic stroke and AMI. Moreover, the strategies devised to enhance resource allocation and patient flow can be embraced by EDs to manage surges in demand, which are anticipated due to population growth and potential new epidemics/pandemics. Furthermore, the methodologies proposed here could be extrapolated to various healthcare settings including hospitalization, surgery, and intensive care. For example, simulation techniques might be employed to predict mortality rates, LOS, and the probability of discharging patients to their homes. DEA could also model patient pathways and treatment alternatives within these services, optimizing the utilization of available resources. Similarly, expanding DEA projects could ensure adherence to healthcare and safety protocols, establishing a foundation for comprehensive data collection and performance analysis in these units.

There are certain limitations to this review. Firstly, the process improvement approaches discussed primarily pertain to the realm of industrial engineering. Considering methodologies beyond this scope, such as clinical management units (CMUs) and clinical-related interventions, could be valuable. Secondly, financial outcomes could be factored into the analysis, potentially constraining the applicability of the described approaches in EDs operating within budget constraints, especially those in low and middle-income countries. Thirdly, despite a carefully implemented and monitored review process, there is a possibility that some relevant studies were inadvertently excluded. Next, valuable insights may have been missed due to the exclusion of the grey literature in the evidence search. Finally, we were unable to address all the other advanced methodologies within MCDM and DEA that have been utilized for diverse objectives. These encompass approaches like DEA fuzzy window analysis [100,101], dynamic DEA [102], the Russell model [103], as well as fuzzy MCDM techniques applied to assess the evaluation dimensions during the COVID-19 pandemic [104,105,106]. Furthermore, well-known MCDM methods such as the technique for order of preference by similarity to ideal solution (TOPSIS), and decision-making trial and evaluation laboratory (DEMATEL) were also applied to categorize significant human error factors in EDs [107,108,109,110,111].

## 4. Conclusions and Future Studies

This narrative review contributes to DMs’ skills in ED performance improvement by highlighting the advantages and drawbacks of DEA. A major benefit is its broad range and all-encompassing approach, effectively bridging the gap between health economists, health services researchers, and DMs in acute care. We identified several crucial aspects to consider when applying DEA studies in stroke and acute care centers. In addition to incorporating these parameters, DEA applications must ensure reproducibility and transparency in both the methodology and results. Researchers are advised to collaborate and work together to enhance the consistency of efficiency measures and maximize the usefulness for consumers. Additional research is required to address the gaps in certain performance measurements, such as incorporating health outcomes as outputs or capital resources as inputs. Furthermore, there is a need to explore the reasons behind productivity changes, their decomposition, and the factors contributing to improved performance in the delivery of ED services. Moreover, developing sensitivity tests is essential to investigate how variations in the DEA model can contribute to uncertainties in the efficiency results. In applying DEA to EDs, there should be more effort to improve the precision of the research outcomes by measuring the sensitivity analysis. Only a subset of articles has utilized methodologies to enhance the model requirements, such as selecting appropriate inputs and outputs, incorporating weight restrictions to account for value judgments in DEA modeling, employing super-efficiency models to handle outlier observations, and thus providing DMs with more dependable information. However, we conducted a comprehensive evaluation of the methodological configurations used in the papers, providing in-depth explanations concerning the models applied, selected inputs and outputs, and all pertinent methodologies. Finally, we investigated multiple approaches to improve DEA’s standing, shifting it from a simple technical application to a strong methodology that can be effectively employed by healthcare managers and decision-makers.

### Future Studies

In future studies, one area of focus within the acute management of stroke will be the application of DEA to benchmark the performance of community hospitals, to enhance the number of stroke patients who meet the eligibility criteria to receive advanced treatment with EVT and therefore require transfer. At the same time, it is necessary to reduce the number of futile transfers of patients who are ineligible for treatment upon arrival. This approach is also suitable for implementation in cases of prompt thrombolysis and PCI for AMI or heart attack. There is also a lack of research studies that apply DEA to evaluate the performance of acute stroke and AMI systems of care. Specifically, DEA can be used to evaluate performances of different EDs to assess the proportion of patients that are treated in both conditions. The proportion of patients that receive treatment is often affected by the population that it serves (e.g., age), the size of the hospital, and the proximity to a large comprehensive center. This provides an opportunity for the application of DEA to provide an efficient frontier that benchmarks this important measure for various hospitals.

## Figures and Tables

**Table 1:** Characteristics of input- and output-oriented basic models.

Characteristics	CCR Input-Oriented	CCR Output-Oriented	BCC Input-Oriented	BCC Output-Oriented
Return to scale (RTS)	CRS	CRS	VRS	VRS
Sign of the inputs	Semi-positive	Semi-positive	Semi-positive	Free
Sign of the outputs	Free	Free	Free	Semi-positive
Type of efficiency	Overall Technical	Overall Technical	Pure Technical	Pure Technical
Surface of envelopment	Piecewise linear	Piecewise linear	Piecewise linear	Piecewise linear
Metric of envelopment	Radial ([0, 1])	Radial ([1, ∞])	Radial ([0, 1])	Radial ([1, ∞])

**Table 2:** Characteristics of the ADD.

Characteristics	Additive Model
RTS	CRS and VRS
Sign of the inputs	Free
Sign of the outputs	Free
Type of efficiency	Mix of both
Surface of envelopment	Piecewise linear
Metric of envelopment	[0, 1]

**Table 3:** Characteristics of SBM model.

Characteristics	SBM Model
RTS	CRS&VRS
Sign of the inputs	Semi-positive
Sign of the outputs	Free
Type of efficiency	Mix of both
Surface of envelopment	Piecewise linear
Metric of envelopment	[0, 1]

## Data Availability

The data used in the study are available from the authors and can be provided upon acceptable requests.

## Abbreviations

Abbreviations	Definitions
ADD	Additive Model
AHP	Analytic Hierarchy Process
AMI	Acute Myocardial Infarction
ANN	Artificial Neural Network
AT	Access Time
BCC	Banker, Charnes, and Cooper
BI	Barthel Index
CCR	Charnes, Cooper, and Rhodes
CRS	Constant Return to Scale
CMUs	Clinical Management Units
CT	Computed Tomography
CTA	CT Angiogram
CTP	CT perfusion
DEMATEL	Decision Making Trial and Evaluation Laboratory
DIDO	Door-In-Door-Out time
DM	Decision-Maker
DEA	Data Envelopment Analysis
DES	Discrete Event Simulation
DMUs	Decision Making Units
DOE	Design of Experiment
DRG	Diagnosis-Related Group
EDs	Emergency Departments
ESCAPE	Endovascular Treatment Compared with Conventional treatment for Patients with Acute Ischemic Stroke
EVT	Endovascular Thrombectomy
FTE	Full-Time Equivalent
GDEA	Generalized Data Envelopment Analysis
GDP	Gross Domestic Product
HCQI	Health Care Quality Indicators
HTP	Health Transformation Programs
HT	Healthcare Time
LOS	Length of Stay
LVO	Large Vessel Occlusion
LWOS	Left Without Being Seen
MCDM	Multi Criteria Decision Analysis
mCTA	multiphase CTA
MI	Myocardial Infarction
ML	Machine Learning
MOLP	Multi-Objective Linear Programming
MPI	Malmquist Productivity Index
MPS	Most Preferred Solution
MRI	Magnetic Resonance Imaging
NINDS	National Institute of Neurological Disorders and Stroke
NNT	Number Needed to Treat
OECD	Organization for Economic Co-operation and Development
OR	Operations Research
OT	Occupational Therapy
OTE	Overall Technical Efficiency
PCI	Percutaneous Coronary Intervention
PPE	Personal Protective Equipment
PTE	Pure Technical Efficiency
PT	Physical Therapy
RTS	Return to Scale
SBM	Slack-Based Measurement
SDEA	Stochastic Data Envelopment Analysis
SE	Scale Efficiency
STEM	Step Method
TE	Technical Efficiency
TFP	Total Factor Productivity
TOPSIS	Technique for Order of Preference by Similarity to Ideal Solution
tPA	Tissue Plasminogen Activator
VRS	Variable Return to Scale
